# Controlled Synthesis of Large-Area Oriented ZnO Nanoarrays

**DOI:** 10.3390/nano14121028

**Published:** 2024-06-13

**Authors:** Haowei Lin, Shibo Xing, Ao Jiang, Mingxuan Li, Qing Chen, Zhenling Wang, Lei Jiang, Huiying Li, Jie Wang, Chenchen Zhou

**Affiliations:** 1 School of Materials Science and Engineering, Henan University of Technology, Zhengzhou 450001, China; xingshibo_123@163.com (S.X.); jascience@163.com (A.J.); limingxuan0722@163.com (M.L.); chenqingmail2024@163.com (Q.C.); 18439411320@163.com (Z.W.); 18336298233@163.com (L.J.); 17513245750@163.com (H.L.); ql050313@163.com (J.W.); z18836520535@163.com (C.Z.); 2 Henan International Joint Laboratory of Nano-Photoelectric Magnetic Materials, Henan University of Technology, Zhengzhou 450001, China

**Keywords:** ZnO, nanoarrays, controlled synthesis, spectral property

## Abstract

Large-area oriented ZnO nanoarrays (including nanowire, nanorod, and nanotube) on ITO glass substrates are synthesized via the simple hydrothermal, electrodeposition, and electrochemical etching approach. The morphology of ZnO nanoarrays is controlled by adjusting the reaction temperature, reaction time, and current density. The scanning and transmission electron microscopy (SEM and TEM) results indicate the successful preparation of large-area oriented ZnO nanoarrays with different types, and the energy-dispersive X-microanalysis spectrum (EDS) and X-ray diffraction (XRD) results confirm that the composition of the obtained nanoarrays is ZnO. More importantly, the as-prepared ZnO nanotube arrays are observed with about a 40% increase in ultraviolet absorption intensity compared to the ZnO nanowire/nanorod arrays, due to having larger specific surface areas. The as-prepared different types of ZnO nanoarrays have great potential for applications in low-cost and high-performance optoelectronic devices.

## 1. Introduction

Zinc oxide is a typical direct bandgap semiconductor with a bandgap width of 3.37 eV. Due to having excellent optoelectronic properties and chemical stability, it has been widely used in the fields of photodetectors, photovoltaic cells, photocatalysts and gas sensors in recent years [1,2,3,4,5,6,7,8,9,10,11,12]. Compared to conventional planar semiconductors, one-dimensional nanostructured semiconductors exhibit some advantages, such as high specific surface areas, good electronic transmission pathways, and light-trapping ability. Moreover, they can not only enhance absorption of high-energy photons, but also promote separation of photo-generated charge carriers [13,14,15]. The nanoarrays, composed of a large number of one-dimensional nanostructures, are beneficial for the preparation of macroscopic optoelectronic nanodevices. Therefore, ZnO nanoarrays have become one of the key materials in the field of optoelectronics [16,17,18,19]. Many methods have been used to synthetize ZnO nanoarrays, such as chemical, electrochemical, and physical deposition techniques [20,21,22,23]. Some studies have shown that the photoelectric properties of ZnO nanoarrays are closely related to the morphology of one-dimensional ZnO nanostructures (including nanowires, nanobelts, nanotubes, etc.) [24]. However, how to control the growth of ZnO nanoarrays with uniform morphology and excellent properties in a low-cost and simple way remains an ongoing challenge in the nanomaterial field. In this work, we present a simple and effective strategy for the controlled synthesizing of different types of large-area oriented ZnO nanoarrays (including nanowire, nanorod and nanotube) with uniform shapes and densities through the organic combination of the hydrothermal, electrodeposition, and electrochemical etching approaches. The entire reaction process can be completed at a relatively low reaction temperature (90 °C) and atmospheric pressure in aqueous solution, with short reaction time and a simple program, as well as good stability and repeatability. In addition, the spectral properties of different types of ZnO nanoarrays have been investigated, and the obtained ZnO nanotube arrays display about a 40% increase in ultraviolet absorption intensity compared to the ZnO nanowire/nanorod arrays. This systematic and efficient method for regulating the morphology of ZnO nanoarrays provides beneficial references for the preparation of ZnO-based heterostructured nanomaterials, and the as-prepared large-area oriented ZnO nanoarrays have great potential for applications in low-cost and high-performance optoelectronic devices.

## 2. Materials and Methods

### 2.1. Materials

Zinc nitrate hexahydrate, zinc acetate dihydrate, lithium hydroxide, and hexamethylenetetramine were purchased from Alfa Aesar Corporation (Ward Hill, MA, USA). Sodium hydroxide, ethylenediamine, hexane, and ethanol were purchased from Shanghai Aladdin Bio-Chem Technology Co., Ltd. (Shanghai, China). These reagents were used directly as purchased. Indium tin oxide (ITO) glass was purchased from Beijing Zhongjing Scientific Instrument Co., Ltd. (Beijing, China). The ZnO nanoarrays were prepared in a home-made electrolytic cell. ZnO nanoarrays were mainly synthesized using the following reactions [25]:

$${\left(CH_{2}\right)}_{6}N_{4}+6H_{2}O↔6HCHO+4NH_{3}↑\;NH_{3}+H_{2}O↔NH_{4}^{+}+{OH}^{-}\;2{OH}^{-}+{Zn}^{2+}↔Zn{\left(OH\right)}_{2}\;Zn{\left(OH\right)}_{2}↔ZnO+H_{2}O$$

### 2.2. Synthesis of ZnO Nanoarrays

#### 2.2.1. Synthesis of ZnO Nanowire Arrays through the Hydrothermal Process

ZnO nanocrystals were firstly synthesized by the sol–gel method through the reaction of lithium hydroxide and zinc acetate dihydrate in ethanol medium, which were separated from the reaction solution by centrifugation and then dispersed in ethanol to form a ZnO nanocrystal colloid. Next, the ZnO nanocrystal colloid was spin-coated 5 times on the ITO glass substrates at the speed of 6000 rpm to form a thin film of ZnO nanocrystals. Next, the ZnO nanowire arrays were synthesized on the obtained ZnO nanocrystal thin film by the hydrothermal method through adjusting the reaction time and temperature. This reaction took place in aqueous solutions of Zinc nitrate hexahydrate and hexamethylenetetramine; its experimental parameters are shown in Table 1.

#### 2.2.2. Synthesis of ZnO Nanorod Arrays by Electrodeposition

In order to synthesize one-dimensional ZnO nanoarrays with better verticality, the electrochemical deposition method was applied to the reaction system. The electrochemical deposition of well-aligned ZnO nanorod arrays was carried out in aqueous solutions of zinc nitrate hexahydrate (0.1 M) and hexamethylenetetramine (0.1 M) with the assistance of an electrochemical workstation (CHI 660E). The ITO glass substrate with a thin film of ZnO nanocrystals and a platinum plate were used as the working electrode and the counter electrode, respectively. Table 2 lists some of the main reaction parameters; the roles of electric current density, reaction temperature, and time in growth of ZnO nanorod arrays were investigated through these reaction systems.

#### 2.2.3. Synthesis of ZnO Nanotube Arrays by Electrochemical Etching

Large-area oriented ZnO nanotube arrays were synthesized through electrochemical etching of the electrodeposited ZnO nanorod arrays, which were prepared under the same conditions as sample N in step 2.3. The electrochemical etching processes were performed in the potentiostatic mode in a homemade glass cell at 60 °C in ethylenediamine aqueous solution (0.1 M); the ITO glass substrates with ZnO nanorod arrays and a platinum sheet were used as the working electrode and counter electrode, respectively. The experimental parameters are shown in Table 3. Subsequently, the obtained ZnO nanotube arrays were rinsed by deionized water several times, and then dried in air at room temperature.

### 2.3. Characterization

The morphological analysis of ZnO nanoarray samples was investigated using field scanning electron microscopy (SEM) (FEI Inspect F50, Hillsborough, OR, USA) and transmission electron microscopy (TEM) (JEOL JEM-2010, Tokyo, Japan). The structural properties were extracted using an X-ray diffractometer (Bruker D8-Advance, Billerica, MA, USA) with a Cu-LFF (λ = 1.54 Å) tube operated at 40 kV–40 mA, employing a scanning rate of 5°/min in the 2θ range from 20°to 80°, and using the database (JCPDS 36-1451 and 65-3170) to identify the phases. Spectral properties were tested by UV-visible spectrophotometer (Shimadzu UV-2600i, Tokyo, Japan).

## 3. Results

**Figure 1:**
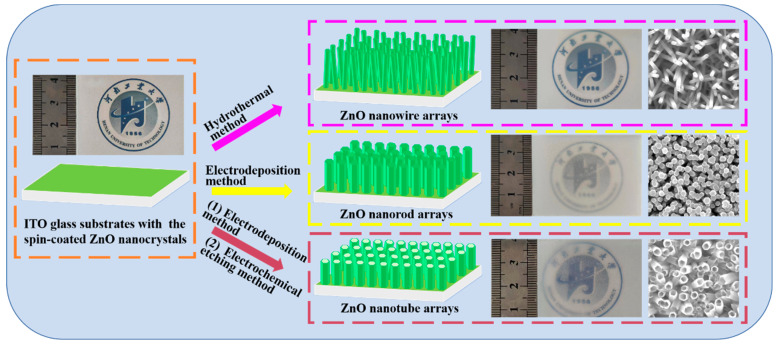
Schematic illustration of controlled synthesis of large-area oriented ZnO nanoarrays.

Different types of large-area ZnO nanoarrays (including nanowire, nanorod, and nanotube) were synthesized on ITO glass substrates by the simple hydrothermal, electrodeposition, and electrochemical etching methods (as shown in Figure 1). As shown in Figure 1, the grey rectangle with the university logo next to the ruler represents a photograph of the ITO glass substrate with the logo, before and after synthesizing ZnO nanoarrays. This logo is the emblem of the author’s institution, Henan University of Technology, which was printed on paper and placed under the ITO glass substrates. After the hydrothermal and electrochemical deposition reactions, the ITO glass exhibits obvious haze due to the formation of ZnO nanoarrays, which reduces the transparency of the ITO glass. Referring to the ruler in this figure, it can be seen that the area of ZnO nanoarrays can reach about 30 cm^2^, which is limited by the spatial size of the home-made reactor. This indicates that these methods can be used to effectively prepare larger areas of zinc oxide nanoarrays in larger reactors. Using the school emblem icon in the figure, it can be seen that the transparency of each ITO glass is relatively uniform, indicating that the diameter and density of the large-area ZnO nanoarrays is basically the same. Combined with the SEM images on the right, it can be clearly observed that compared to the ZnO nanowire arrays, the diameter and density of the nanorod arrays is larger, resulting in a decrease in the transparency of the corresponding glass sheets. After the ZnO nanorod arrays were etched into nanotube arrays, the transparency of the corresponding glass sheets increased.

### 3.1. Microscopic Morphology Analysis of ZnO Nanowire Arrays Synthesized by the Hydrothermal Method

#### 3.1.1. The Influence of Reaction Temperature on the Morphology of ZnO Nanowire Arrays

**Figure 2:**
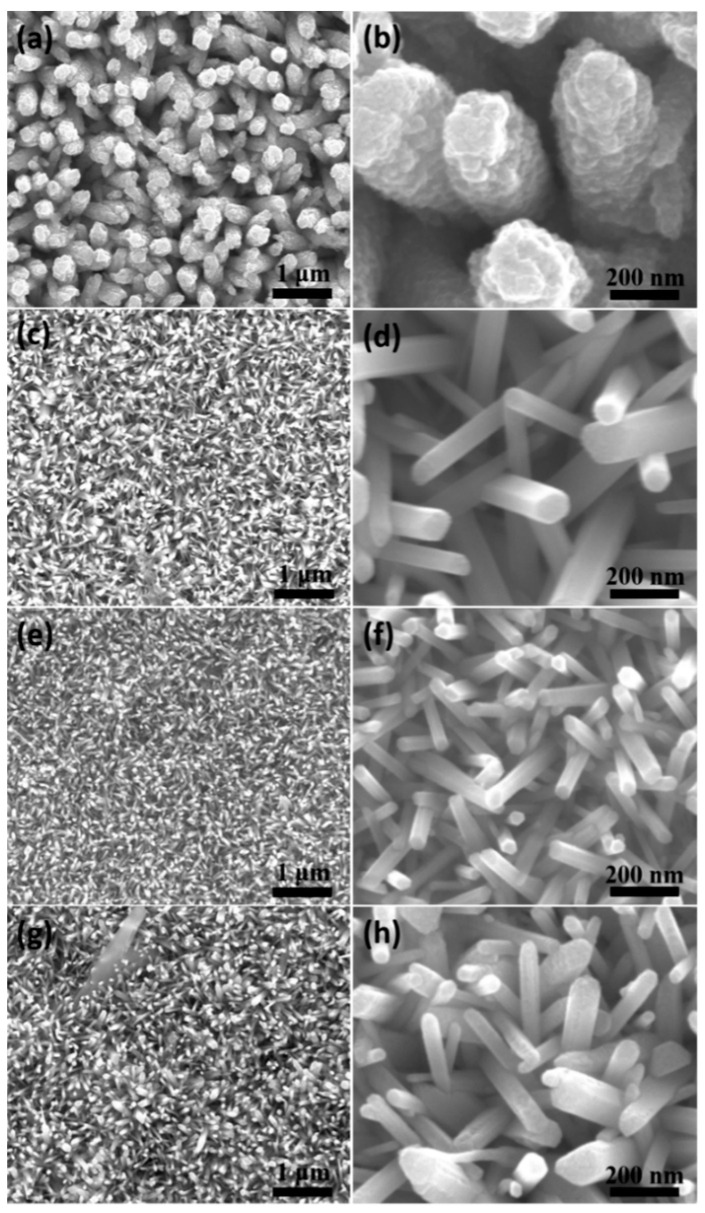
SEM images of ZnO nanowire arrays prepared by the hydrothermal method under different temperature conditions: (**a**,**b**) 80 °C, 4 h; (**c**,**d**) 85 °C, 4 h; (**e**,**f**) 90 °C, 4 h; (**g**,**h**) 95 °C, 4 h.

Due to the significant influence of temperature on the crystallization of inorganic substances, the effect of temperature on the microstructure of ZnO nanowire arrays was first investigated. Figure 2 shows the morphology and size of the ZnO nanorod arrays prepared by the hydrothermal method at different reaction temperatures under the same reaction time (4 h). As shown in Figure 2a,b, when the reaction temperature was only 80 °C, the morphology of the ZnO nanowire array showed an irregular stacking shape with many uneven small particles on the surface. The hexagonal surface profile of the ZnO nanowires was not obvious, which may be attributed to the lower reaction temperature and shorter reaction time, resulting in insufficient growth momentum, incomplete maturation, and imperfect crystal growth of ZnO crystals.

**Figure 3:**
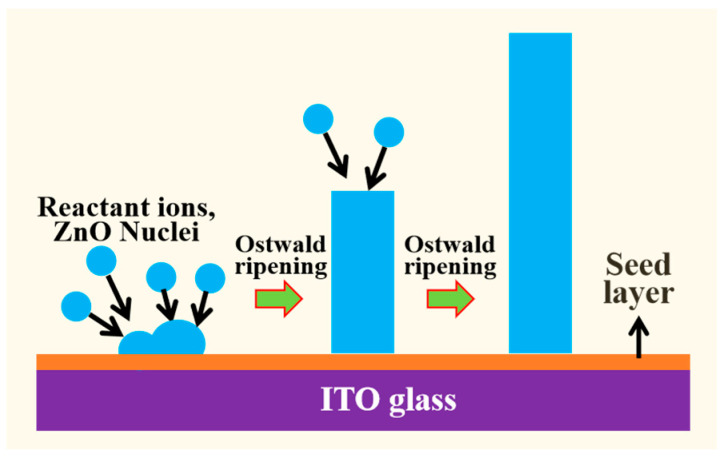
Schematic diagram of ZnO nanowire growth.

Figure 3 shows the schematic diagram of growth of ZnO nanowires on the ITO glass substrates. Firstly, the reagent ions started to react rapidly and nucleate, then they attached to the seed layer on the surface of ITO glass and grew into nanoparticles. Next, the nanoparticles would undergo Ostwald ripening, where smaller nanocrystals dissolved while larger nanocrystals continued to grow, resulting in the more uniform and wider size distribution of nanocrystals, ultimately forming an ordered array of nanowires [26,27]. When the reaction temperature rose to 85 °C (Figure 2c,d), the small particles on the surface of the ZnO nanowires disappeared, mainly due to the intensification of atomic thermal motion with increasing temperature. Some atoms had higher energy than the interface barrier of the small particles, and these high-energy atoms could cross the interface of the grain, causing the interface to fuse and disappear. Thus, the surface of the ZnO nanowires exhibited the hexahedral structure of hexagonal wurtzite. However, their diameter distribution was uneven, which was related to the insufficient driving force for growth of ZnO nanowire arrays at this temperature. Figure 2e,f shows the morphology of the ZnO nanowire array at a reaction temperature of 90 °C. It can be observed that the diameter of ZnO nanowires is uniform and about 62 nm, and their shape is a typical regular hexagon. In Figure 2g,h, it can be seen that as the reaction temperature rises to 95 °C, the diameter distribution of the ZnO nanowire arrays exhibited obvious non-uniformity. Mainly due to the increase in thermal entropy of atoms within the crystal at higher temperatures, the orientation of ZnO molecules arranged along the habit plane decreases, thereby increasing the disorder of ZnO nanowire arrays growth. In summary, 90 °C is the most suitable growth temperature for ZnO nanowire arrays.

#### 3.1.2. The Influence of Reaction Time on the Morphology of ZnO Nanowire Arrays

**Figure 4:**
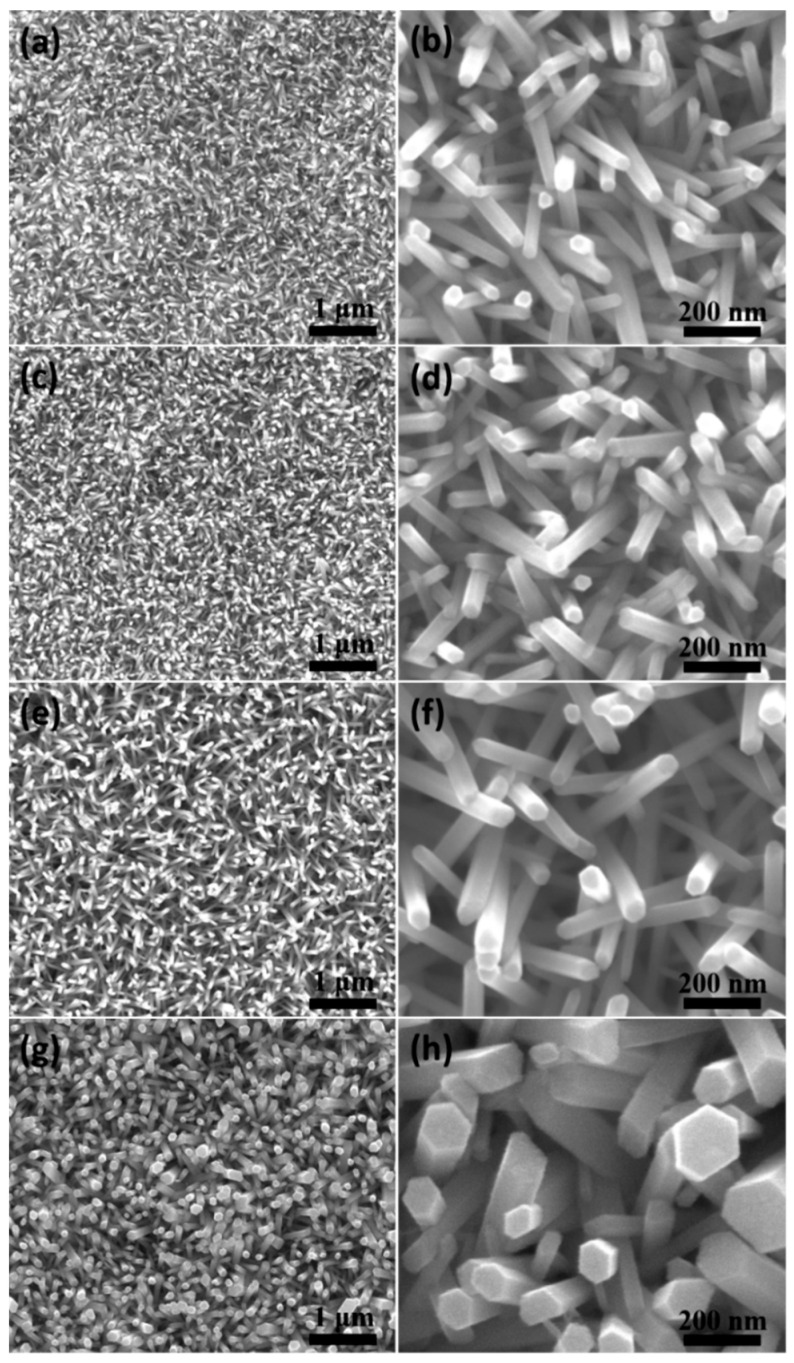
SEM images of ZnO nanowire arrays prepared by the hydrothermal method under different reaction times: (**a**,**b**) 90 °C, 2 h; (**c**,**d**) 90 °C, 4 h; (**e**,**f**) 90 °C, 6 h; (**g**,**h**) 90 °C, 8 h.

Figure 4 reveals the influence of reaction time on the morphology of ZnO nanowire arrays at a reaction temperature of 90 °C. It can be seen that as the reaction time extended, the diameter distribution of the ZnO nanowire arrays became more uniform, and the diameter gradually increased. The average diameter gradually increases from 34 nm (2 h), to 46 nm (4 h), to 62 nm (6 h). However, when the reaction time was extended to 8 h, the diameter uniformity of the ZnO nanowire arrays significantly decreased. As the reaction time further increased, the difference in growth rate of different ZnO nanowires increased, resulting in a larger diameter of the nanowires with faster growth rates. Therefore, prolonged growth actually led to an increase in the non-uniformity of the diameter distribution of ZnO nanowire arrays. In summary, large-area ordered ZnO nanowire arrays with uniform size can be synthesized by the hydrothermal method at 90 °C for 6 h.

**Figure 5:**
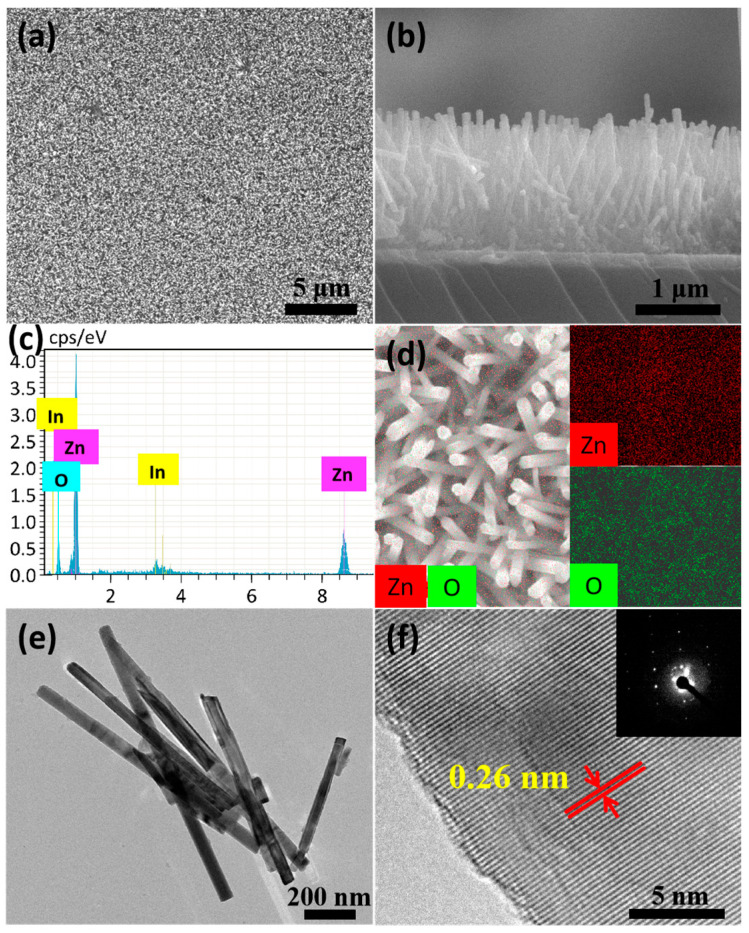
SEM and TEM images of ZnO nanowire arrays prepared by the hydrothermal method at 90 °C for 6 h: (**a**) top view of large-area ZnO nanowire arrays; (**b**) side view of ZnO nanowire arrays; (**c**) energy-dispersive X-microanalysis spectrum (EDS) of the ZnO nanowire arrays; (**d**) element mapping of the ZnO nanowire arrays; (**e**) TEM image of several typical ZnO nanowires; (**f**) HRTEM image of the ZnO nanowire; the distance between the red lines sandwiched within red arrows indicates the interplanar spacing; the inset is the selective area electron diffraction pattern (SAED) taken from the nanowire.

Figure 5 shows more microscopic morphologies of ZnO nanowire arrays prepared by the hydrothermal method at 90 °C for 6 h. Figure 5a reveals a large number of uniform ZnO nanowires at a lower magnification, implying that this reaction condition is suitable for the synthesis of large-area ZnO nanowire arrays. The size and density of these ZnO nanowires are relatively uniform, resulting in a similar height of the ZnO nanowire array from a side view in Figure 5b. The result of EDS element analysis shows that the nanowire arrays are mainly composed of the elements Zn and O (Figure 5c). Quantitative analysis indicates that the atomic ratio of Zn and O is about 1:1. The In peak of the spectrum is assigned to the ITO glass substrate. The uniform dispersion of the elements Zn (red) and O (green) in the as-prepared nanowires can be seen clearly in Figure 5d. These results strongly demonstrate that the composition of the as-prepared nanowires is ZnO. As shown in Figure 5e,f, TEM and HRTEM results present further structural characterizations of the as-prepared nanowires. Some typical ZnO nanowires can be seen in Figure 5e, and they have a diameter of about 62 nm and a length of about 1.5 μm, which corresponds well to the above SEM images. The lattice spacing of 0.26 nm of the nanowires was clearly observed in Figure 5f, which is in good agreement with the interplanar distance of the (002) direction parallel to the hexagonal wurtzite phase of ZnO [28]. The selective area electron diffraction pattern (SAED) of the as-prepared nanowires in the inset of Figure 5f indicates that the crystalline structure of these nanowires is close to single-crystal.

### 3.2. Microscopic Morphology Analysis of ZnO Nanorod Arrays Synthesized by the Electrodeposition Method

#### 3.2.1. The Influence of Current Density on the Morphology of ZnO Nanorod Arrays

**Figure 6:**
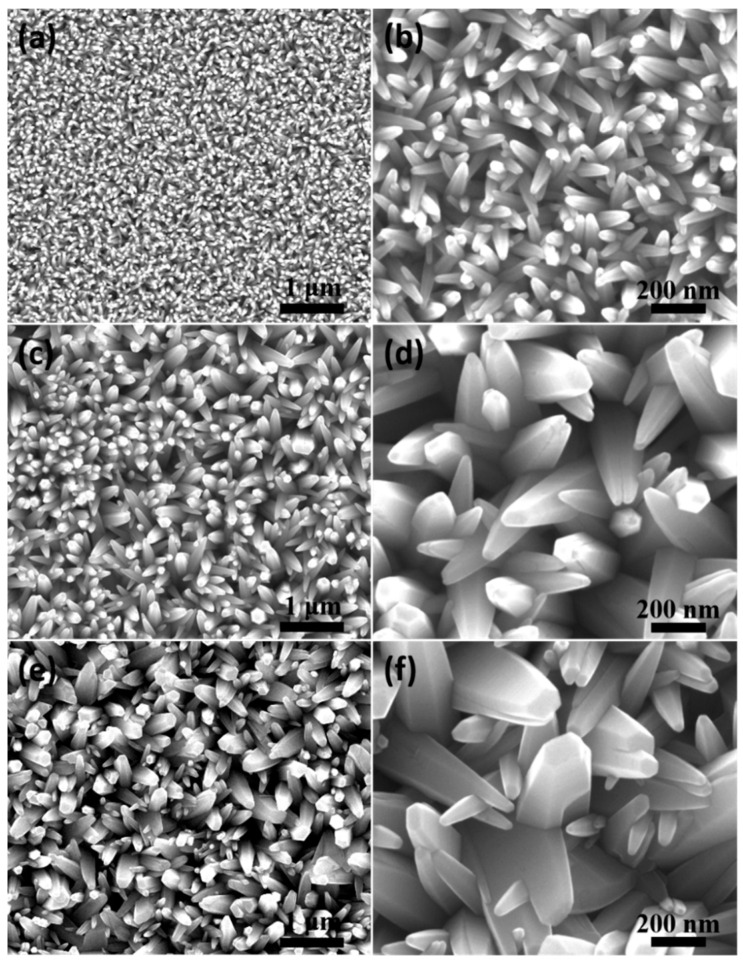
SEM images of ZnO nanorod arrays synthesized by the electrodeposition method at different current densities under the same reaction temperature (90 °C) and reaction time (1 h): (**a**,**b**) 0.1 mA/cm^2^; (**c**,**d**) 0.25 mA/cm^2^; (**e**,**f**) 0.45 mA/cm^2^.

Figure 6 presents the microscopic morphology of ZnO nanorod arrays synthesized by the electrodeposition method at different current densities under the same reaction temperature (90 °C) and time (1 h). Compared to ZnO nanowire arrays synthesized by the hydrothermal method mentioned above, the diameter of the as-prepared nanorod arrays was significantly larger, and they presented conical structures. The difference from the aforementioned hydrothermal method was that an external electric field was applied in the reaction system, which provided a driving force for the migration of reaction ions, resulting in a higher electromigration efficiency of reactants in the reaction system than that of free diffusion, thereby significantly accelerating the growth rate of nanorods compared to hydrothermal methods [29]. In addition, with the increase in current density from 0.1 mA/cm^2^ to 0.45 mA/cm^2^, the disorder of ZnO nanorod array growth was enhanced, and the diameter difference between different nanorods increased. The main reason was that with enhancement of the applied electric field, the preferred growth trend of ZnO nanorods with higher maturation degree became more pronounced. Obviously, the diameter uniformity of ZnO nanorod arrays is relatively best under the current density of 0.1 mA/cm^2^.

#### 3.2.2. The Influence of Reaction Time on the Morphology of ZnO Nanorod Arrays

**Figure 7:**
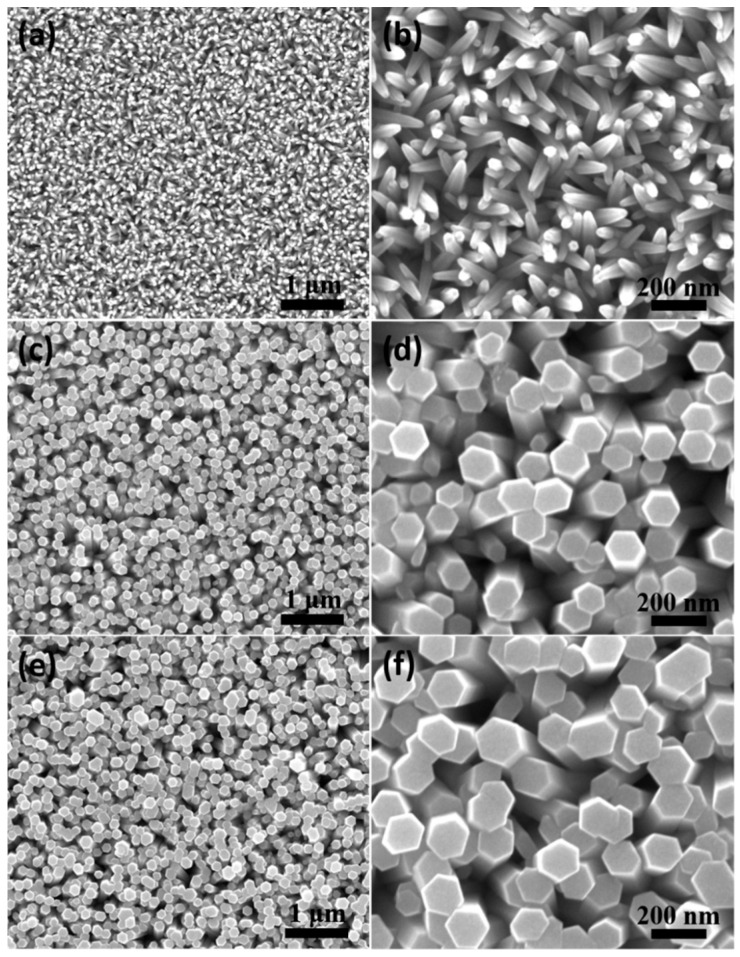
SEM images of ZnO nanorod arrays prepared by the electrodeposition method at different reaction time under the same reaction temperature (90 °C) and current density (0.1 mA/cm^2^): (**a**,**b**) 1 h; (**c**,**d**) 3 h; (**e**,**f**) 5 h.

The effect of reaction time on the morphology of ZnO nanorod arrays can be seen in Figure 7. Through comparison of these SEM images, it can be found that as the reaction time extended, the shape of the top of the ZnO nanorods gradually changed from a conical shape to a typical hexagonal shape, and the diameter distribution became more uniform. When the reaction time was extended to 5 h, the diameters of the ZnO nanorods were the most uniform and about 148 nm. In fact, under the condition of external electric field assistance, an electric field enhancement effect occurs at the top of the ZnO nanorod array, which led to a certain electric field bending effect around the top of the ZnO nanorod array. This was beneficial for the tip of the ZnO nanorod array to adsorb more zinc ions in the reaction solution to form ZnO [30].

**Figure 8:**
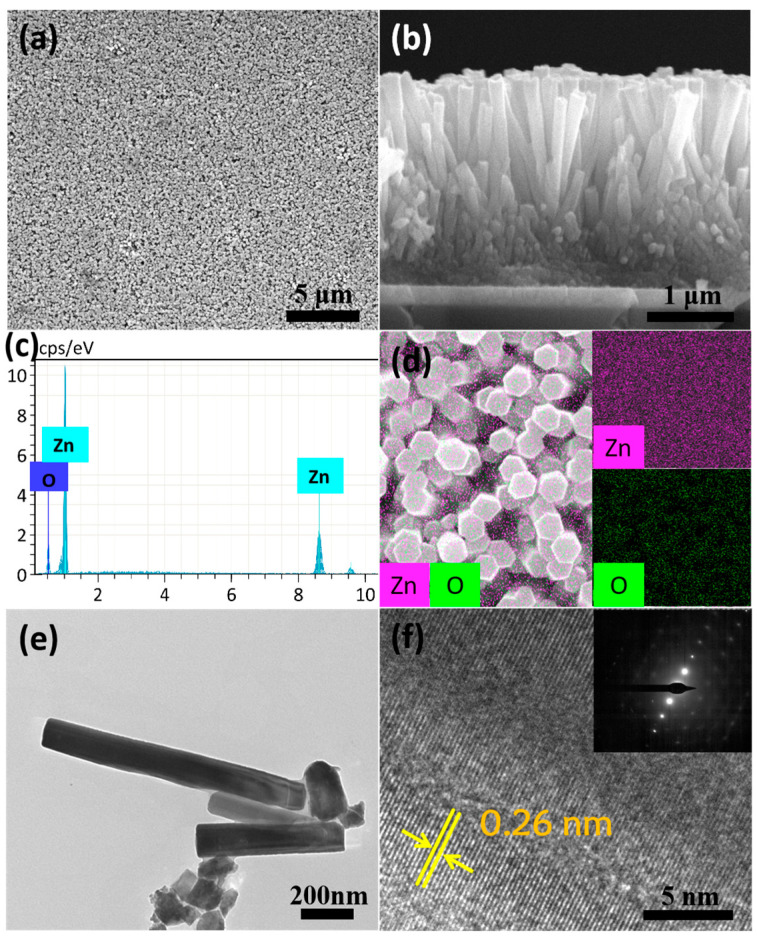
SEM and TEM images of ZnO nanoroad arrays synthesized by the electrodeposition method at 90 °C for 5 h under the current density of 0.1 mA/cm^2^: (**a**) top view of large-area ZnO nanoroad arrays; (**b**) side view of ZnO nanoroad arrays; (**c**) EDS of the ZnO nanoroad arrays; (**d**) element mapping of the ZnO nanoroad arrays; (**e**) TEM image of several typical ZnO nanorods; (**f**) HRTEM image of the ZnO nanoroad; the distance between the yellow lines sandwiched within yellow arrows indicates the interplanar spacing; the inset is the selective area electron diffraction pattern (SAED) taken from the nanorod.

Further microscopic morphologies of the ZnO nanorod arrays prepared by the electrodeposition method at 90 °C for 5 h under the current density of 0.1 mA/cm^2^ are shown in Figure 8. In Figure 8a, the SEM image of a large number of nanorods at a lower magnification looks like a carpet with consistent thickness and density. From the side view of Figure 8b, it can be observed that ZnO nanorods have good verticality, with a length of about 1.8 μm. In in Figure 8c,d, the EDS result shows that the constituent elements of the nanorod arrays are only Zn and O, the uniformly dispersion of the elements Zn (pink) and O (green) in the nanorod arrays confirms that the composition of the as-prepared nanorods is ZnO. Figure 5e displays some typical ZnO nanorods with a diameter of about 145 nm and a length of about 1.5 μm, which corresponds well with the SEM image of Figure 8b. The HRTEM image in Figure 8f presents the lattice spacing of 0.26 nm of the nanorod, which is also in good agreement with interplanar distance of the (002) direction parallel to the hexagonal wurtzite phase of ZnO. The SAED of the inset in Figure 8f shows the almost single-crystal structure of the as-prepared ZnO nanorod arrays.

### 3.3. Microscopic Morphology Analysis of ZnO Nanotube Arrays Prepared by the Electrochemical Etching Method

**Figure 9:**
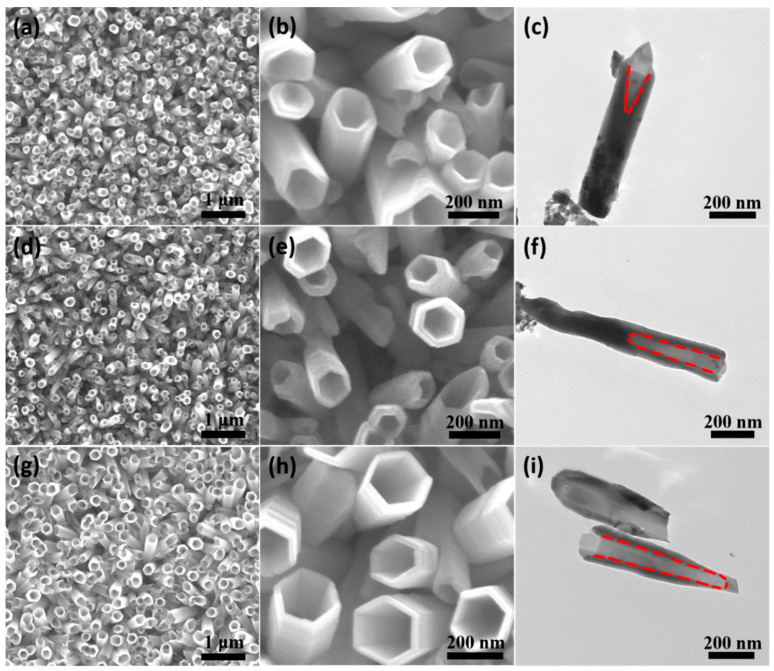
SEM and TEM images of ZnO nanotube arrays prepared by the electrochemical etching method at different reaction times: (**a**–**c**) 0.5 h; (**d**–**f**) 1 h; (**g**–**i**) 1.5 h. The part between the red dashed lines is the nanotube structure.

Figure 9 shows the microscopic morphology of ZnO nanotube arrays prepared by the electrochemical etching method at different reaction times using the ZnO nanorod arrays synthesized above as a template. From SEM images of Figure 8a, b, it can be seen that ZnO nanorods were preferentially etched from the top center into partial nanotubes, which is intuitively confirmed by the TEM image of Figure 8c. The lighter-colored area marked by the red dashed line is the nanotube structure, while the darker and denser part is the nanorod structure. As shown in Figure 9d–i, as the reaction time increases from 0.5 h to 1.5 h, the ZnO nanorods continued to be etched mainly along the axial direction, resulting in a more complete structure of the nanotubes. ZnO nanorods were etched into nanotubes through the following chemical reactions [31]:

$$H_{2}N{\left(CH_{2}\right)}_{2}NH_{2}+2H_{2}O↔H_{2}N{\left(CH_{2}\right)}_{2}NH_{4}^{2+}+2OH^{-}\;ZnO+2OH^{-}\to ZnO_{2}^{-}+H_{2}O\;2ZnO+4OH^{-}=2Zn{\left(OH\right)}_{2}+O_{2}↑$$

Under the effect of an electric field, on the one hand, when ZnO nanorod arrays were subjected to positive potential, the holes moved towards the tops of the nanorods, resulting in a space charge effect around the top surface, attracting hydroxide ions to react with zinc oxide molecules. The potential induces the migration of hydroxide ions to the top of the zinc oxide nanorods and increases their transfer rate, which accelerates the selective etching of zinc oxide nanotubes. On the other hand, the selective electrochemical etching of ZnO nanorods is related to the surface energy of each crystal plane. Due to the maximum surface energy in the [0001] direction, the ZnO nanorods were preferentially etched along the C-axis [32]. These factors together led to preferential etching at the center of the ZnO nanorods, forming a tubular structure.

**Figure 10:**
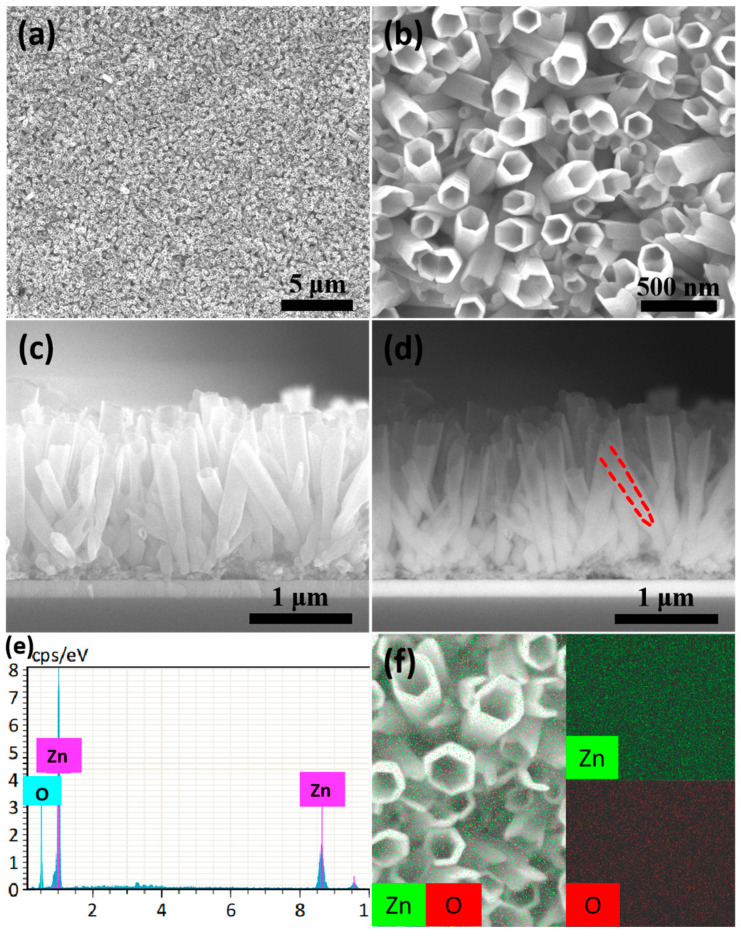
SEM images of ZnO nanotube arrays synthesized by the electrochemical etching method at a reaction time of 1.5 h: (**a**,**b**) top view of the large-area ZnO nanotube arrays under low and high magnification; (**c**) second electronic image of the side view of the ZnO nanotube arrays; (**d**) background scattered electron image of the side view of the ZnO nanotube arrays; the part between the red dashed lines is the nanotube structure; (**e**) EDS of the ZnO nanotube arrays; (**f**) element mapping of the ZnO nanotube arrays.

Figure 10 presents more information of ZnO nanotube arrays synthesized by the electrochemical etching method at a reaction time of 1.5 h. Massive uniformly shaped nanotubes are seen in the Figure 10a,b, indicating the successful preparation of large-area ordered ZnO nanotube arrays. In Figure 10c, the second electronic image of the side view shows clearly that the obtained nanotubes still have good verticality and basically consistent lengths. Moreover, the background scattered electron image of the side view of the ZnO nanotube arrays more intuitively displays the nanotube structure, such as the light-colored part marked by the red dashed line in Figure 10d, indicating that the nanorods had been etched thoroughly into nanotubes. The EDS result of Figure 10e reveals that the obtained nanotube arrays are composed of Zn and O elements, which exhibit a uniform distribution on the obtained arrays seen from element mapping of Figure 10f, indicating that the composition of the nanotube arrays is ZnO.

### 3.4. XRD Studies of the ZnO Nanoarrays

**Figure 11:**
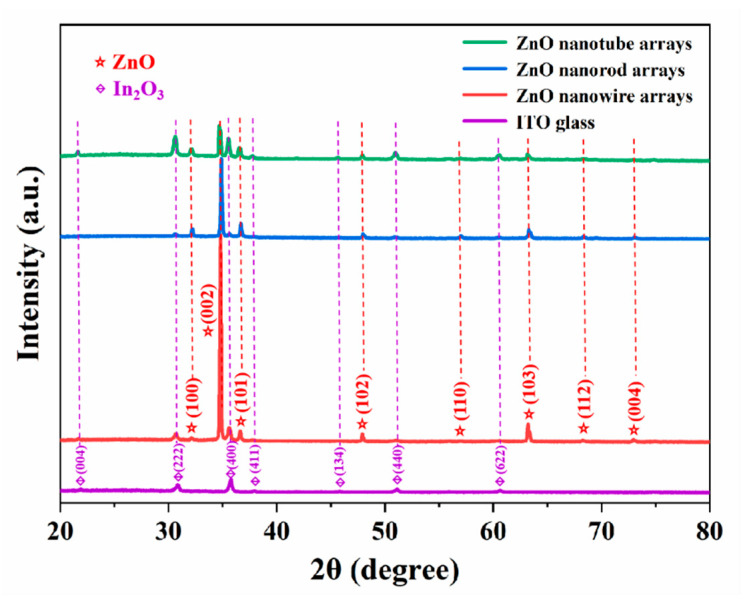
XRD patterns of the ZnO nanowire arrays (red), ZnO nanorod arrays (blue), and ZnO nanotube arrays (green).

The XRD patterns of the obtained different ZnO nanoarrays are presented in Figure 11. The characteristic absorption peaks (purple annotation) at diffraction angles of 22.2°, 31.4°, 36.5°, 37.4°, 45.8°, 51.2°, and 61.3° belong to the (004), (222), (400), (411), (134), (440), and (622) diffraction planes of ITO glass substrates, respectively [33]. In the XRD patterns of the ZnO nanowire, nanorod, and nanorod arrays, the diffraction peaks (red annotation) are identified to match the hexagonal ZnO crystal structure with the structure of wurtzite, the name and number of the space group of which are P6_3_mc and 186, respectively [34]. Among them, there is a strong peak at 34.5°, corresponding to the (002) diffraction crystal plane of ZnO, indicating that the ZnO nanorods exhibit a preferential growth orientation along the C-axis direction [0001]. Compared to ZnO nanowire and nanorod arrays, the diffraction peak of the (002) crystal plane of ZnO nanotube arrays at 34.5° was significantly weakened, indicating that ZnO nanorods were preferentially etched along the direction with the highest surface energy (corresponding to the C-axis) during electrochemical etching.

### 3.5. UV-Vis Spectrum Studies of the ZnO Nanoarrays

**Figure 12:**
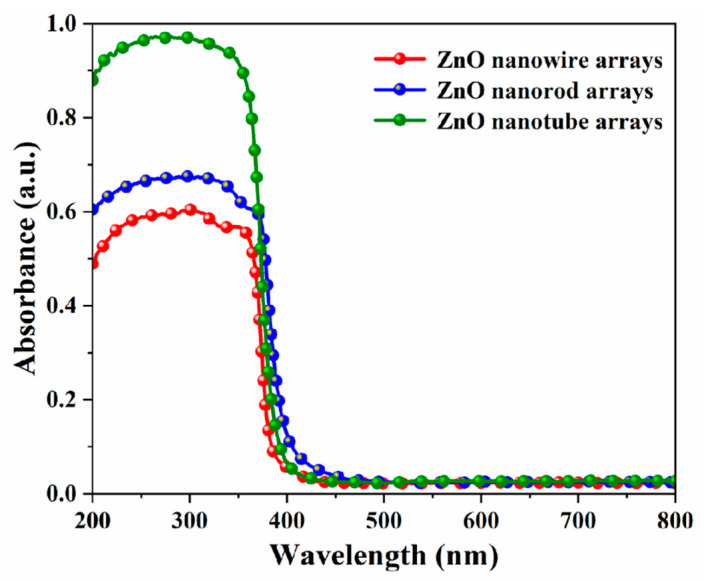
Normalized UV-visible absorption spectra of the different ZnO nanoarrays.

Figure 12 presents the UV-visible absorption spectra of different ZnO nanoarrays. It can be seen that these nanoarrays absorb in the UV region with a band edge of ~390 nm, which is consistent with the typical UV absorption characteristics of ZnO [34]. Compared to the ZnO nanowire arrays (hydrothermal method, red line), the absorption of the ZnO nanorod arrays (electrodeposition method, blue line) and the ZnO nanotube arrays (electrochemical etching method, green line) in the ultraviolet region was significantly enhanced in sequence. Due to their higher density and good verticality, the ZnO nanorod arrays exhibited stronger ultraviolet absorption compared to the ZnO nanowire arrays. Electrochemical etching enabled nanotubes to have larger specific surface areas, which was beneficial for light absorption and could promote light reflection and refraction, resulting in about a 40% increase in ultraviolet absorption intensity of ZnO nanotube arrays compared to the ZnO nanowire/nanorod arrays.

## 4. Conclusions

In summary, this work presents a typical fabrication of large-area oriented ZnO nanoarrays (including nanowire, nanorod, and nanotube) on ITO glass substrates by the simple hydrothermal, electrodeposition, and electrochemical etching methods. Through comparing and analyzing the effects of reaction temperature, reaction time, and current density on the morphology of ZnO nanoarrays, it was found that the uniform nanowire arrays with a diameter of about 62 nm and a length of about 1.5 μm could be prepared by the hydrothermal method at 90 °C for 6 h, the ZnO nanorod arrays with better verticality with a diameter of about 145 nm and a length of about 1.8 μm could be synthesized by the electrodeposition method at 90 °C for 5 h under the current density of 0.1 mA/cm^2^, which could be further electrochemically etched into the uniform ZnO nanotube arrays by reacting for 1.5 h. Excitingly, compared to the ZnO nanowire/nanorod arrays, the as-prepared ZnO nanotube arrays exhibited about a 40% increase in ultraviolet absorption intensity due to having larger specific surface areas. These results indicate the great application potential of the as-prepared ZnO nanoarrays on macroscale fabrication of low-cost and high-performance optoelectronic devices.

## Figures and Tables

**Table 1:** Experimental parameters used in the hydrothermal process for synthesis of ZnO nanowire arrays.

Sample Number	Concentration of Zn(NO_3_)_2_·6H_2_O (M)	Concentration of C_6_H_12_N_4_ (M)	Reaction Temperature (°C)	Reaction Time (h)
A	0.1	0.1	80	4
B	0.1	0.1	85	4
C	0.1	0.1	90	4
D	0.1	0.1	95	4
E	0.1	0.1	90	2
F	0.1	0.1	90	6
G	0.1	0.1	90	8

**Table 2:** Experimental parameters used in the electrodeposition of ZnO nanorod arrays.

Sample Number	Electric Current Density (mA/cm^2^)	Reaction Temperature (°C)	Reaction Time (h)
H	0.1	90	1
I	0.25	90	1
J	0.45	90	1
K	0.1	90	3
L	0.1	90	5

**Table 3:** Experimental parameters used in the electrochemical etching of ZnO nanotube arrays.

Sample Number	Voltage (V)	Reaction Temperature (°C)	Reaction Time (h)
M	−0.6	60	0.5
N	−0.6	60	1
O	−0.6	60	1.5

## Data Availability

Data are contained within the article.
